# 4286 The Relationship Between Tinnitus-Related Distress and PTSD Symptoms Among Post 9/11 Veterans with Posttraumatic Headache

**DOI:** 10.1017/cts.2020.150

**Published:** 2020-07-29

**Authors:** John Moring, Casey Straud, Donald Penzien, Patricia Resick, Alan Peterson, Carlos Jaramillo, Blessen Eapen, Cindy McGeary, Jim Mintz, Willie Hale, Don McGeary

**Affiliations:** 1 University of Texas Health Science Center San Antonio; 2 Wake Forest School of Medicine; 3 Duke University School of Medicine; 4 UT Health San Antonio; 5 South Texas VA Healthcare System; 6 UT San Antonio

## Abstract

OBJECTIVES/GOALS: Military personnel are at significantly greater risk for developing tinnitus, due to increased exposure to acoustic trauma. Many psychiatric disorders are common among individuals with chronic tinnitus, including posttraumatic stress disorder (PTSD). Although tinnitus and PTSD are clearly different, research supports the notion of shared mechanisms between both disorders. First, there are overlapping symptoms between tinnitus-related distress and PTSD, including irritability, distorted cognitions, persistent negative emotional states, diminished interests in activities, exaggerated startle response, sleep disturbance, concentration problems, and hypervigilance. Second, tinnitus and PTSD are highly comorbid with one another, whereas 34% of veterans with tinnitus also carry a PTSD diagnosis. Third, those with both disorders are significantly more emotionally impaired compared to those with tinnitus and any other psychiatric disorder. Lastly, neuroimaging research has shown similar regions within the auditory vigilance network are implicated among those with tinnitus, and separately, among combat PTSD patients, suggesting shared neurobiological mechanisms between both disorders. Though we are aware that tinnitus and comorbid PTSD presents as a significantly greater clinical concern, the relationship between tinnitus-related distress and PTSD symptomotology it is still unknown. Canonical correlation analyses will be conducted to examine the relationship between tinnitus-related distress and PTSD among veterans as a part of a larger clinical trial for posttraumatic headache. Results of the study will shed light on the relationship between tinnitus-related distress and PTSD, and may suggest a different phenotype for those with both disorders. Researchers and clinicians will further understand and conceptualize the relationships among the cognitive, emotional, and behavioral symptoms associated with tinnitus and PTSD, both individually and conjointly. METHODS/STUDY POPULATION: Baseline data (N = 112) from a larger clinical trial examining the effectiveness of two different psychotherapies for the alleviation of posttraumatic headache was examined. The primary aim of this project was to evaluate the relationship between tinnitus-related distress and PTSD based on the eight subscale scores of the Tinnitus Functional Index (TFI) and the four scales of the Clinician Administered PTSD Scale for the DSM-5 (CAPS-5), respectively. To address this aim, canonical correlation analysis was used where the tinnitus-related symptom subscales made up one variable set and PTSD symptom subscales made up the second variable set. First, we evaluated the overall model fit based on Wilks Lambda to determine if the two variable sets were related at the *p* < .05 level. Next, we evaluated the canonical correlations (comparable to an eigenvalue) for each canonical dimension to determine the required number of significant canonical dimensions (or latent constructs) that were necessary to understand the association between the two variable sets. Finally, the standardized canonical coefficients, which are analogous to regression coefficients, evaluate the magnitude of variate relationships and determine which subscales best describe significant canonical dimensions. RESULTS/ANTICIPATED RESULTS: Prior to the canonical correlation analysis, total score descriptive statistics and subscale score zero-order correlations were carried out. The CAPS-5 total score was 33.24 (SD = 9.39) and the TFI total score was 50.81 (SD = 21.88) in this sample. Interpretation of the zero-order correlations indicated that TFI Relaxation subscale was the only tinnitus-related subscale moderately associated with a PTSD subscale (i.e., Reexperiencing, *r* = .35). Canonical correlation omnibus model fit analysis via the Wilks Lambda overall multivariate test indicated that the tinnitus variable set was significantly associated with the PTSD variable set, *F* = 1.55, *p* = .04. Evaluation of the canonical correlations indicated that one dimension was significant in explaining the relationship between the two variable sets and accounted for 25% of the overall variance, *F* = 1.55, *p* < .04, R^2^ = .249. Standardized canonical coefficients indicated that the PTSD subscales Reexperiencing (*b* = 0.64) and Negative Alterations in Cognition and Mood (*b* = 0.55) were the most representative of the identified canonical dimension. In terms of the TFI, the Relaxation (*b* = 1.28) and Sleep (*b* = 0.72) subscales appeared to be most related to the canonical dimension. The TFI subscales Auditory Difficulty (*b* = −0.30) and Quality of Life (*b* = 0.30) also appeared to be related the canonical dimension to a lesser degree. DISCUSSION/SIGNIFICANCE OF IMPACT: Findings support prior research suggesting particularly deleterious functional outcomes among individuals with comorbid tinnitus and PTSD. Results of this study suggest a latent variable that can explain the unique experience of individuals with both disorders. This latent variable consists of two PTSD constructs: Reexperiencing traumatic events (i.e., flashbacks, nightmares, intrusive memories), and Negative Alterations in Cognition and Mood (i.e., self- and other-blame, strong negative feelings, loss of interest, feeling distant). This latent variable also consists of two tinnitus-related constructs: Sleep (i.e., trouble falling and staying asleep, peaceful sleep) and Relaxation (i.e., ability to relax, enjoyment of peace and quiet). Auditory Difficulty (i.e., hear clearly, understand people) and Quality of Life (i.e., social activities, relationships, difficulty performing tasks) also contributed to the latent variable, but to a lesser degree. It is suggested that the constellation of symptoms related to the latent variable is a Dysphoric Factor, unique to individuals with PTSD, tinnitus, and posttraumatic headache. It may be necessary to incorporate different techniques into existing evidence-based treatments for both tinnitus and PTSD, for optimal symptom improvement.

